# The impact of personal relative deprivation on aggression over time

**DOI:** 10.1080/00224545.2018.1549013

**Published:** 2018-12-13

**Authors:** Tobias Greitemeyer, Christina Sagioglou

**Affiliations:** University of Innsbruck

**Keywords:** Aggression, personal relative deprivation, contagion, longitudinal data

## Abstract

Being at a disadvantage and perceiving this predicament to be unfair are at the core of the experience of personal relative deprivation. Previous research has shown that personal relative deprivation is associated with interpersonal aggression. The present longitudinal study extended these investigations by examining the impact of personal relative deprivation on aggression over time. In fact, personal relative deprivation at Time 1 was associated with reported aggression at Time 2 even when controlling for the impact of aggression at Time 1. As a secondary goal, we aimed to show that the effect of personal relative deprivation (i.e., increased aggression) may spread through the participant’s social network. Egocentric networking data showed that individuals who perceive their friends as being personally deprived are more aggressive and that this relationship statistically holds when taking the individual’s level of personal relative deprivation into account. Limitations of this approach are discussed.

Inequality in income and wealth among their inhabitants is high in most modern societies. For example, U.S. Americans in the top 1% earn over 40 times more than the bottom 90%, and the 8.6% world’s wealthiest individuals own 85.6% of the global wealth (Inequality.org, 2018). The unequal distribution of access to resources may have a serious impact on how people who (accurately) perceive themselves to be worse off than others think, feel, and behave. For example, whereas being better off than others increases emotional well-being, having less than others has the opposite effect (e.g., Wills, 1981). Moreover, perceiving that one is relatively disadvantaged to others and that this predicament is unfair may lead to the experience of personal relative deprivation, which then evokes feelings of anger and resentment (Smith, Pettigrew, Pippin, & Bialosiewicz, 2012). The present study examined the long-term impact of personal relative deprivation on reported aggression. It was predicted that the experience of personal relative deprivation would increase aggression (while controlling for initial levels of aggression). As a secondary goal, we tested whether the effect of personal relative deprivation (i.e., aggression) would spread through the participant’s social network.

## The relationship between personal relative deprivation and aggression

Abundant evidence suggests that the perception to be at a disadvantage has important cognitive, affective, and behavioral consequences (for a meta-analysis, Smith et al., 2012). For example, the experience of personal relative deprivation is associated with negative cognitions and depressive symptoms (Beshai, Mishra, Meadows, Parmar, & Huang, 2017), with poorer physical and mental health (Mishra & Carleton, 2015), and increased mortality (Sloggett & Joshi, 1994). Most relevant for the present research, personal relative deprivation has been shown to be associated with affective hostility, aggressive behavior, antisocial conduct, and criminal outcomes (Baron, 2003; DeCelles & Norton, 2016; Greitemeyer & Sagioglou, 2017; Mishra & Novakowski, 2016).

These latter findings are fully in line with recent theorizing that describes the relative deprivation experience (Smith & Pettigrew, 2014; Smith et al., 2012). According to the theory of relative deprivation, an individual’s objective position in a social hierarchy instigates an interpersonal comparison between the individual and similar other persons. If the individual comes to the conclusion that the other persons are better off and that the disadvantage is undeserved, s/he likely responds with anger and resentment, which in turn instigates aggressive action.

Previous studies into the relationship between personal relative deprivation and aggression have either employed a cross-sectional correlational or an experimental design. What is missing is evidence that personal relative deprivation has long-lasting effects on aggression. To address this gap, the present study employed a longitudinal design and examined the cross-lagged relationships between personal relative deprivation and aggression over two time points. Although the predictions for our longitudinal study are the same as for previous cross-sectional correlational or experimental studies, a longitudinal design provides unique insights in that it is the only methodology that can examine changes over a longer period of time. For example, experimental work showed that personal relative deprivation led to higher levels of aggressive affect, which then instigated aggressive action (Greitemeyer & Sagioglou, 2017). However, inasmuch as aggressive affect and action were immediately measured after the deprivation manipulation, aggression may fade within a short period of time and thus the effect of personal relative deprivation is of little practical importance. Moreover, long-lasting effects of personal relative deprivation on aggression would provide strong evidence for the theory of relative deprivation.

Whereas the main focus of our study was on the long-term effects of personal relative deprivation, we also examined the impact of an individual’s objective and subjective socioeconomic status (SES) on reported aggression. An individual with low objective SES has little access to material resources such as income, wealth, and educational attainment, whereas the opposite is true for an individual with high SES (Côté, 2011). Objective measures of SES are typically moderately related to people’s subjective perceptions of their social standing. Previous research has shown that objective measures of SES are negatively associated with hostile inclinations (for a review, Gallo & Matthews, 2003). Likewise, individuals with low rather than high subjective SES are more prone to aggressive responding (Chen, Zuo, & Zhao, 2018; Greitemeyer & Sagioglou, 2016, 2018, in press). Because an individual’s (both objective and subjective) SES should be unlikely to change much over time, we did not expect that SES has effects on changes in aggression, but, rather, that SES is associated with aggression at Time 1. Hence, we assessed participant’s objective and subjective SES at Time 1 only.

## The contagious effects of aggression

As noted above, a secondary goal of our study was to examine whether individuals are more aggressive the more deprived they perceive their social contacts to be. It is a well-known finding that aggression and violence spread through social networks (for a review, Dishion & Tipsord, 2011). That is, being the victim of aggressive acts and even the mere observation of aggression toward others typically leads to increased aggression in the future. Hence, we expected that an individual who experiences personal relative deprivation would be more aggressive and that this increased aggression then should increase aggression in individuals with whom the individual is connected.

## The present research

Our main goal was to examine the long-term impact of personal relative deprivation on aggression. As in previous research (Baron, 2003; DeCelles & Norton, 2016; Greitemeyer & Sagioglou, 2017; Mishra & Novakowski, 2016), personal relative deprivation should be negatively associated with reported aggression. Extending these previous investigations, we tested whether personal relative deprivation would be associated with changes in aggression over time. It was expected that personal relative deprivation at Time 1 would predict reported aggression at Time 2 even when controlling for the impact of reported aggression at Time 1. Because participant sex and age could be associated with both, personal relative deprivation (Bettencourt & Miller, 1996; Callan, Kim, & Matthews, 2015b) and aggression (Archer, 2004; Liu, Lewis, & Evans, 2013), we also controlled for the impact of these variables. Moreover, objective and subjective SES have been shown to be associated with personal relative deprivation and aggression (e.g., Chen et al., 2018; Gallo & Matthews, 2003; Greitemeyer & Sagioglou, 2016, 2018, in press), so we included these variables in our analyses as well.

We were also interested in whether increased aggression due to the experience of personal relative deprivation could spread through social networks. To test our reasoning, we employed egocentric networking analyses (Stark & Krosnick, 2017). In an egocentric networking approach, participants provide self-reports but also report on how they perceive their social contacts (called “friends” in the following). In some ego-centered network surveys, participants also indicate, for every pair of friends, whether these two individuals know each other. However, other studies (e.g., Greitemeyer, 2018; Mötteli & Dohle, in press) abstained from assessing ties between friends. We also did not assess the nature of the relationship between participant’s friends, because this was not essential for the hypothesis that participants are more aggressive if they perceive their friends to be personally deprived. It was predicted that perceived friends’ personal relative deprivation would be associated with increased aggression in the participants and that this relationship would remain significant when controlling for participant level of personal relative deprivation. To ensure that it is indeed aggression that spreads across the participant’s social network, we also ran a mediation analysis testing whether friends’ level of aggression would mediate the impact of perceived friends’ personal relative deprivation on participant’s reported aggression. Our study received ethical approval from the Internal Review Board for Ethical Questions in Science of the University of Innsbruck (55/2017).

## Method

Participants were citizens of the United States, recruited on Amazon Mechanical Turk (MTurk). MTurk provides an online participant pool that is much more representative of the U.S. population than are university samples (Paolacci & Chandler, 2014). There were no data exclusions, and all participants were run before any analyses were performed. Because it was unknown how many participants would follow the invitation to complete the questionnaire at Time 2, no a priori power analyses were conducted. Instead, we decided to run a large number of participants so that statistical power can be ensured. At Time 1, there were 2502 participants (1376 females, 1126 males; mean age = 35.7 years, *SD *= 11.8). Of these, 980 participants (522 females, 458 males; mean age = 38.9 years, *SD *= 12.5) completed the questionnaire at Time 2. Time 1 and Time 2 were 6 months apart.

Education, income, and subjective SES were only assessed at Time 1. Ten participants did not finish high school, 280 participants completed high school, 847 participants completed some college, 986 participants obtained a Bachelor’s degree, 306 participants had a Master’s degree, and 73 participants had a PhD degree. To assess income, participants were given eight categories to estimate their annual household’s income (cf. Piff, Kraus, Côté, Cheng, & Keltner, 2010). The categories were: (1) < $15,000, (2) $15,001–$25,000, (3) $25,001–$35,000, (4) $35,001–$50,000, (5) $50,001–$75,000, (6) $75,001–$100,000, (7) $100,001–$150,000, (8) > $150,000. Participants’ responses were close to a normal distribution (category 1: 6.8%, category 2: 11.4%, category 3: 13.2%, category 4: 17.5%, category 5: 21.9%, category 6: 14.8%, category 7: 10.8%, category 8: 3.9%). Education and income were significantly correlated, *r* = .35, *p* < .001, so these measures were standardized and averaged into one overall measure of objective SES.

Subjective SES was assessed using the MacArthur Ladder (Adler, Epel, Castellazzo, & Ickovics, 2000). Participants were shown a picture of a 10-rung ladder and asked to imagine that this ladder represents where people stand in the United States. At the top of the ladder are the people who are the best off—those who have the most money, the most education, and the most respected jobs, whereas at the bottom are the people who are the worst off—who have the least money, the least education, and the least respected jobs or no jobs. Participants were asked to type the number of the rung where they think they stand at this time of their life relative to other people in the United States.

Participants’ self-reported personal relative deprivation was assessed using a scale that has been successfully employed in previous research (Callan, Shead, & Olson, 2011). There were five items. Sample items: “I feel deprived when I think about what I have compared to what other people like me have” and “I feel privileged compared to other people like me” (recoded). The scale ranged from 1 (*strongly disagree*) to 7 (*strongly agree*). Internal consistency was α = .84.

To assess participants’ reported aggressive behavior, participants were asked to indicate how often they had shown the respective behavior in the past six months (e.g., Krahé & Möller, 2010). There were 10 items (α = .89). Sample items: “I have hit another person” and “I have played one person off against another”. The scale ranged from 1 (*never*) to 5 (*very often*).

Participants then learned that they would be asked questions about 3 people they feel closest to (e.g., friends, coworkers, neighbors, relatives). For each friend, they reported the level of aggression (αs between .90 and .91), perceived SES, and personal relative deprivation (αs between .88 and .90), employing the same questions as for themselves. Responses to the three friends were then averaged. The questionnaire included some additional questions (i.e., participant’s violent video game exposure) that will be reported elsewhere.^1^ Finally, participants were thanked and asked what they thought this experiment was trying to study, but none noted the exact hypothesis that their friends’ deprivation would affect their own level of aggression. At Time 2, the same measures of relative deprivation and reported aggression were employed. Reliabilities were similar to Time 1 (personal relative deprivation: α = .85; reported aggressive behavior: α = .90; friends’ perceived deprivation: αs between .85 and .89; friends’ aggression: α = .90 for each of the three friends).

## Results

For Time 1 (*N* = 2502), descriptive statistics and intercorrelations of all measures are shown in Table 1. For Time 2 (*N* = 980), descriptive statistics and intercorrelations of all measures are shown in Table 2. Note that participants’ objective and subjective SES were only assessed at Time 1.

**Table 1.** Means, Standard Deviations, and Bivariate Correlations (Time 1, *N* = 2502)

	*M*	*SD*	1	2	3	4	5	6	7
1. Participant sex	-	-							
2. Participant age	35.7	11.8	.06**						
3. Participant objective SES	.00	0.82	-.01	.05*					
4. Participant subjective SES	4.96	2.91	.03	.03	.34***				
5. Participant personal deprivation	3.42	1.28	-.04	-.04*	-.29***	-.29***			
6. Participant aggression	1.38	0.52	-.08***	-.21***	-.04*	-.00	.14***		
7. Friends’ perceived deprivation	3.45	1.16	-.00	-.07***	-.15***	-.14***	.43***	.17***	
8. Friends’ aggression	1.39	0.49	-.04*	-.17***	-.11***	-.02	.15***	.69***	.31***

Note: * *p* < .05, ** *p* < .01, *** *p* < .001

Participant sex was coded 1 = male, 2 = female.

**Table 2.** Means, standard deviations, and bivariate correlations (Time 2, *N* = 980).

	*M*	*SD*	1	2	3	4	5
1. Participant sex	-	-					
2. Participant age	39.0	12.5	.07*				
3. Participant personal deprivation	3.46	1.38	-.05	-.08*			
4. Participant aggression	1.30	0.45	-.09**	-.17***	.16***		
5. Friends’ perceived deprivation	3.32	1.17	-.04	-.08*	.46***	.17***	
6. Friends’ aggression	1.33	0.44	-.06	-.14***	.15***	.74***	.28***

Note: * *p* < .05, *** *p* < .001

Participant sex was coded 1 = male, 2 = female.

### Attrition analyses

We first examined whether the sample of individuals who completed both questionnaires differed from those who completed only the first questionnaire. We received data from 980 individuals who completed both questionnaires, whereas 1483 individuals completed only the first questionnaire. For the remaining 39 individuals, a Worker ID-based match with the second sample failed and so it is unknown whether they only completed the first questionnaire or both questionnaires. These latter participants were excluded from the attrition analyses. The analyses showed that individuals who took part at Time 1 but did not take part at Time 2 (*M* = 1.43, *SD* = 0.53) reported to be more aggressive than individuals who took part at both time points (*M* = 1.32, *SD* = 0.50), *t*(2461) = 5.08, *p* < .001, *d* = 0.21. They were also older (*M* = 40.3, *SD* = 14.3 vs. *M* = 35.3, *SD* = 12.8), *t*(2461) = 9.00, *p* < .001, *d* = 0.37. No significant differences were observed for participants’ personal relative deprivation, participant sex, objective SES, and subjective SES. Importantly, the key relationships between participants’ personal relative deprivation and their reported aggression, *r*(980) = .14, *p* < .001, *r*(1483) = .15, *p* < .001, as well as participants’ reported aggression and perceived friends’ relative deprivation, *r*(980) = .17, *p* < .001, *r*(1483) = .17, *p* < .001, were very similar for participants who completed both questionnaires or just the first.

### The relationship between SES and reported aggression

Subjective SES was moderately associated with objective SES. Unexpectedly, subjective SES was not associated with reported aggression. In contrast, subjective SES was negatively associated with personal relative deprivation. Objective SES was negatively associated with reported aggression, but the relationship was small. Objective SES was also negatively associated with personal relative deprivation.

### The relationship between personal relative deprivation and reported aggression

As hypothesized, personal relative deprivation was positively associated with reported aggression. We also examined whether the impact of personal relative deprivation on reported aggression would hold when controlling for participant sex, age, objective SES, and subjective SES. In a multiple regression, personal relative deprivation, participant sex (coded 1 = male, 2 = female), age, objective SES, and subjective SES were used as predictors for reported aggression. The overall regression was significant, *F*(5, 2496) = 35.23, *p* < .001, *R*^2^ = .07. Most importantly, personal relative deprivation was still significantly associated with reported aggression, *t* = 6.68, β = .14, *p* < .001. Participant sex, *t* = 3.23, β = −.06, *p* = .001, and age, *t* = 10.28, β = −.20, *p* < .001, were also significant predictors. Objective SES was not associated with reported aggression, *t* = 0.47, β = −.01, *p* = .639, whereas subjective SES received a significant regression weight, *t* = 2.10, β = .04, *p* = .036. Unexpectedly, however, the relationship was positive.

**Figure 1.**
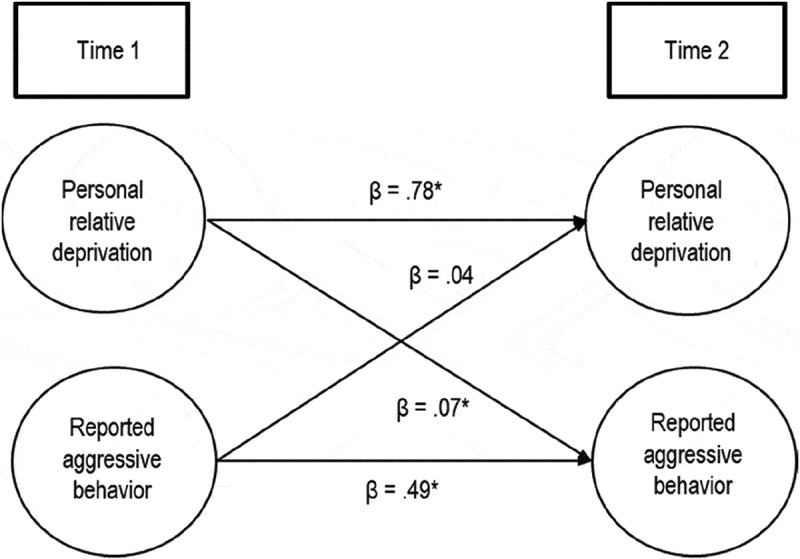
The longitudinal relation between personal relative deprivation and reported aggressive behavior. Standardized coefficients are shown (* denotes a significant path).

Before examining the impact of personal relative deprivation on reported aggression over time, we aimed to show that the relationship among the items for Time 1 and Time 2 measures were structurally similar (i.e., measurement invariance). To do so, we calculated measurement invariance through a sequence of invariance models, which we then tested for difference (Vandenberg & Lance, 2000). For both personal relative deprivation and aggressive behavior, scalar measurement invariance (Steenkamp & Baumgartner, 1998) was established. The metric measurement invariance model did not significantly differ from the scalar invariance model; for relative deprivation: χ^2^ = 5.20, *df* = 4, *p* = .268; for aggressive behavior: χ^2^ = 2.73, *df *= 9, *p* = .974. Given that measurement invariance could be established, a cross-lagged regression analysis was then performed on the data. Personal relative deprivation and reported aggression at Time 1 were used as predictors for reported aggression at Time 2 (*N* = 980). The overall regression was significant, *F*(2, 977) = 165.88, *R*^2^ = .25, *p* < .001. As can be expected, reported aggression showed high stability, *t* = 17.55, β = .49, *p* < .001. Importantly, personal relative deprivation at Time 1 significantly predicted reported aggression at Time 2, *t* = 2.46, β = .07, *p* = .014. This effect remained significant when controlling for participant sex, age, objective SES, and subjective SES, *t* = 2.01, β = .06, *p* = .044. The reverse effect that reported aggression at Time 1 affects personal relative deprivation at Time 2 when controlling for personal relative deprivation at Time 1, as well as participant sex, age, objective SES, and subjective SES, was not significant, *t* = 1.57, β = .03, *p* = .117. It thus appears that personal relative deprivation leads to changes in aggression, whereas the effect of behaving aggressively on the experience of deprivation is less reliable. The bidirectional relation between personal relative deprivation and reported aggression is illustrated in Figure 1.

### Egocentric social networking analysis

At Time 1 (*N* = 2502), participants’ and friends’ perceived relative deprivation as well as their level of reported aggression, respectively, were positively associated (Table 1). That is, participants perceived their friends to be similar to them. Importantly, participants’ reported aggression was also associated with perceived friends’ relative deprivation. It was then examined whether perceived friends’ relative deprivation is still associated with participants’ reported aggression when controlling for the impact of participants’ personal relative deprivation. To this end, perceived friends’ relative deprivation and participants’ personal relative deprivation were used as predictors for participants’ reported aggression. The overall regression was significant, *F*(2, 2499) = 44.86, *R*^2^ = .04, *p* < .001. Most importantly, the impact of perceived friends’ relative deprivation remained significant, *t* = 6.29, β = .14, *p* < .001. Participants’ personal relative deprivation also significantly predicted participants’ reported aggression, *t* = 3.73, β = .08, *p* < .001. When also controlling for participant sex, age, objective SES, and subjective SES, perceived friends’ relative deprivation remained a significant predictor, *t* = 5.91, β = .13, *p* < .001.

**Figure 2.**
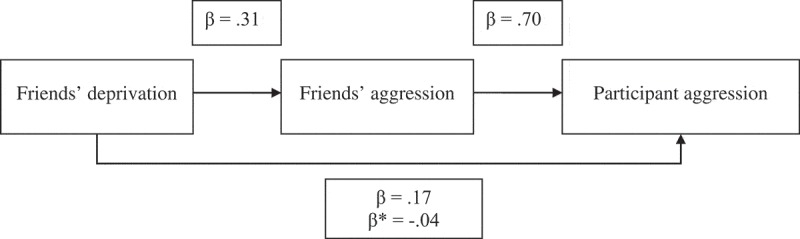
Mediation of the impact of the friends’ perceived deprivation on participants’ reported aggression by friends’ aggression. All paths are significant. β* = the coefficient from friends’ deprivation to participant aggression when controlling for friends’ aggression.

We then examined whether friends’ level of aggression would account for the relationship between perceived friends’ relative deprivation and participant’s reported aggression (when controlling for the impact of participants’ personal relative deprivation). A bootstrapping analysis (with 5.000 iterations) showed that the impact of friends’ level of aggression was significant (point estimate: .74, 95% CI = [.71, .77]). Participants’ personal relative deprivation remained positively associated with participants’ reported aggression (point estimate: .03, 95% CI = [.01, .04]). In contrast, perceived friends’ relative deprivation was now negatively associated (point estimate: −.03, 95% CI = [−.05, −.02]), suggesting that friends’ level of aggression serves as a suppressor. The indirect effect was significantly different from zero (point estimate: .09, 95% CI = [.08, .11]). This mediation effect—based on regression coefficients—is shown in Figure 2. Moreover, when we employed participant sex, age, objective SES, and subjective SES as covariates, a bootstrapping analysis showed that the mediation effect remained significant (point estimate: .09, 95% CI = [.08, .10]).

Finally, we ran a regression analysis where perceived friends’ relative deprivation and participants’ reported aggression at Time 1 were used as predictors for participants’ reported aggression at Time 2 (*N* = 980). The overall regression was significant, *F*(2, 977) = 165.31, *R*^2^ = .25, *p* < .001. Perceived friends’ relative deprivation at Time 1 significantly predicted participants’ reported aggression at Time 2, *t* = 2.59, β = .07, *p* = .010. Participants’ reported aggression at Time 1 also received a significant regression weight, *t* = 17.37, β = .49, *p* < .001. However, the impact of perceived friends’ relative deprivation at Time 1 was no longer significant when controlling for participants’ relative deprivation at Time 1, *t* = 1.68, β = .05, *p* = .094.

## Discussion

The present research provides the first evidence that personal relative deprivation has long-lasting effects on reported aggression. By employing a longitudinal design, we could show that personal relative deprivation leads to increased aggression over time. Importantly, by controlling for initial levels of aggression, a host of alternative explanations can be ruled out (e.g., confounding variables). The present research thus extends previous studies (Baron, 2003; DeCelles & Norton, 2016; Greitemeyer & Sagioglou, 2017; Mishra & Novakowski, 2016) that have employed cross-sectional correlational or experimental designs. Taken all these studies together, the picture is quite clear that being at a disadvantage and perceiving this predicament to be unfair increases aggressive action. Notably, the present study is the first that documents that these effects do not fade within a short period of time but can be observed even 6 months later. Our findings thus provide key support for the theory of relative deprivation (Smith & Pettigrew, 2014; Smith et al., 2012) that the experience of personal relative deprivation may lead to aggressive action.

Future research may address the psychological mechanisms of how precisely personal relative deprivation instigates aggressive action over time. One likely candidate are hostile emotional reactions. According to the theory of relative deprivation (Smith et al., 2012), the experience of being worse off than others leads to anger and resentment. These hostile feelings then evoke aggressive action. It is thus conceivable that personal relative deprivation is only a distal determinant of aggressive behavior, whereas hostile feelings are the proximal determinants. It should be noted, however, that the experience of personal deprivation cannot only lead to hostile emotional reactions, but also to fear (for example), which then evokes different behavioral outcomes (i.e., exit, Osborne, Smith, & Huo, 2012). Future research may also explore the role of stable personality characteristics (e.g., negative affectivity, sensitivity to justice, neuroticism) that might contribute to the relationship between personal deprivation and aggression.

Please also note that we assessed a general tendency to feel deprived relative to others, rather than the evaluation of one initial event (e.g., an adverse social comparison) that then may lead to aggression. Given that this tendency is relatively stable, we can only speculate about how exactly feelings of deprivation cause aggression some months later. As postulated by social comparison theory (Festinger, 1954), individuals often compare themselves to others in their daily life to learn where they stand. Some of these interpersonal comparisons involve other persons that stand considerably higher in a social hierarchy. If the disadvantage is perceived to be unjust, the experience of personal relative deprivation follows. The accumulation of these deprivation experiences then leads to the general feeling that other persons are better off, which in turn appears to increase aggressive inclinations. It may thus be the accumulation of unfavorable comparisons that lead to increased feelings of anger and resentment, which eventually evoke aggressive behavior in individuals.

It is also noteworthy that individuals cannot only experience personal relative deprivation but also group-based deprivation when a social group to which they belong is deprived. Whereas personal relative deprivation should be associated with interpersonal aggression, group-based deprivation should be associated with intergroup hostility (Smith et al., 2012). In the present research, we focused on the consequences of personal relative deprivation, but we acknowledge that the impact of group-based deprivation on aggression over time also warrants empirical research.

Please also recall that we did not assess who the target of participants’ aggressive behavior was. This is important insofar as the model of relative deprivation (Smith et al., 2012) assumes that the impact of personal deprivation on aggression should be most pronounced when taking action is directed toward redressing the deprivation experience (e.g., when the target is perceived as responsible for one’s deprivation). However, inasmuch as research into displaced aggression has shown that individuals tend to react aggressively against innocent targets after they had been provoked by another person (Marcus-Newhall, Pedersen, Carlson, & Miller, 2000), the experience of personal deprivation should also increase aggressive inclinations against uninvolved targets. Other relative deprivation theorists (e.g., Crosby, 1976; Feather, 2015; Folger, 1987) propose other dimensions, such as feasibility and the likelihood of amelioration, that also affect who is most likely to be a target of an individual’s experienced personal deprivation. Future research should examine more closely who is most likely the target of aggression that is elicited by personal deprivation experiences.

Importantly, personal deprivation does certainly not always lead to aggression. For example, if other responses appear to be more effective to redress one’s deprivation or if individuals believe that the disadvantage can be changed (e.g., people believe they can climb up the social ladder, Sagioglou, Forstmann, & Greitemeyer, in press), they will likely withhold their aggressive impulses.

Both subjective and objective SES were negatively associated with the experience of relative deprivation. Unexpectedly, however, participant’s subjective SES was not significantly associated with their reported aggression. Moreover, the relationship between objective SES and reported aggression was very small. These findings are in contrast to previous research that has consistently shown that both objective and subjective SES are associated with different measures of aggression (e.g., Chen et al., 2018; Gallo & Matthews, 2003; Greitemeyer & Sagioglou, 2016, 2018, Greitemeyer & Sagioglou, in press). It should be noted that the present study employed a self-report measure of aggression, whereas many of the previous studies employed behavioral measures of aggression (although not all of them). Given that most individuals are prone to positive self-evaluations, participants may have underestimated the degree to which they behaved aggressively. In fact, the overall mean of our aggression scale was very low (see Table 1). It is thus conceivable that the impact of participants’ SES on their reported aggression was rather minimal because of the relatively small range of responses for the aggression measure.

Notably, the relationship between subjective SES and reported aggression was even positive when controlling for participants’ personal relative deprivation. Likewise, recent work has addressed the unique contributions of personal relative deprivation, subjective SES, and objective SES in the context of health outcomes and prosocial behaviors (Callan, Kim, Gheorghiu, & Matthews, 2017; Callan, Kim, & Matthews, 2015a). Interestingly, Callan et al. (2017) found that subjective SES was not associated with helping behavior, but when controlling for the impact of perceived relative deprivation, subjective SES was a negative predictor. Future work may further explore to what extent personal relative deprivation and subjective SES function as mutual suppressors in their prediction of prosocial and antisocial behavior.

A secondary goal of our study was to examine whether the impact of personal relative deprivation (i.e., increased aggression) would spread through the participant’s social network. In fact, we found that participants who perceived their friends as being personally deprived reported to be more aggressive. Importantly, this relationship statistically held when controlling for participants’ level of personal relative deprivation. Moreover, mediation analyses showed that the level of friends’ perceived aggression accounted for the relationship between perceived friends’ personal relative deprivation and participants’ reported aggression. It thus appears that a person’s experience of personal relative deprivation may indeed affect others that are connected to the person. Because the deprived person becomes more aggressive, others also become more aggressive.

It should be noted, however, that the longitudinal effect of perceived friends’ relative deprivation on participants’ reported aggression at Time 2 was no longer significant when controlling for participants’ reported aggression at Time 1 as well as their personal relative deprivation at Time 1. Moreover, an important limitation of egocentric network data is the reliance on participants’ perception of their social network (for a recent review of egocentric network analysis, Perry, Pescosolido, & Borgatti, 2018). It may well be that participants’ perceptions of their friends do not correspond to their friends’ self-perceptions. On the other hand, it is rather likely that people’s behavior is more strongly affected by how they perceive their friends rather than their friends’ objective qualities. Nevertheless, future research that employs sociocentric network data where information about the friends is provided by the friends themselves is needed before one can come to the conclusion that aggression due to the experience of personal relative deprivation spreads through social networks.

As in previous research that employed egocentric networking analyses (e.g., Greitemeyer, 2018; Mötteli & Dohle, in press), participants received no further instructions whom to list as a friend other than that they should feel close to them. Hence, we did not examine whether these friends are a source of participants’ personal deprivation (i.e., the friends are better off) or may also feel resentful because they themselves experience to be worse off than others. Both scenarios may contribute to the participants’ increased aggression, but due to a different underlying reason. Future research would thus benefit from exploring the relationship between participants and their friends in more detail. Having better off close friends may increase unfavorable social comparisons and thus feelings of deprivation and hostile emotional reactions, which could be further reinforced if these close friends are perceived as similar others (see Smith et al., 2012).

A final limitation involves our use of cross-lagged regression analyses to test our hypotheses. These analyses appear to confound between- and within-person associations, so parameter estimates should be interpreted with caution (Berry & Willoughby, 2017; Hamaker, Kuiper, & Grasman, 2015). However, alternative methods typically need more than two waves of data to meaningfully disaggregate individual-level change from between-person change (cf. Osborne, Milojev, & Sibley, 2017).

## Conclusion

As noted in the introduction, the experience of personal relative deprivation has important negative intrapersonal consequences (e.g., depressive symptoms, poorer physical and mental health, and increased mortality). Moreover, it has negative interpersonal effects, in that aggressive behavior is increased. As the present study shows, these effects can occur on a long-term basis. Any policy changes that decrease the experience of disadvantage, which could be as diverse as reducing inequality or increasing social mobility, would thus benefit not only the people that are at the bottom of the social hierarchy, but their social environment would benefit as well.

## Data Availability

The data described in this article are openly available in the Open Science Framework at https://osf.io/jp8ew/

## Note

1. Participant’s violent video game exposure was positively associated with both personal relative deprivation, *r*(2502) = .08, *p* < .001, and reported aggression, *r*(2502) = .15, *p* < .001. However, the relationship between personal relative deprivation and reported aggression, *r* = .14, was relatively unaffected when controlling for violent video game exposure, *r* = .13.

## Open Scholarship

This article has earned the Center for Open science badges for Open Data and Open Materials through Open Practices Disclosure. The data and materials are openly accessible at https://osf.io/jp8ew/
